# Biological Functions of *ilvC* in Branched-Chain Fatty Acid Synthesis and Diffusible Signal Factor Family Production in *Xanthomonas campestris*

**DOI:** 10.3389/fmicb.2017.02486

**Published:** 2017-12-12

**Authors:** Kai-Huai Li, Yong-Hong Yu, Hui-Juan Dong, Wen-Bin Zhang, Jin-Cheng Ma, Hai-Hong Wang

**Affiliations:** ^1^Guangdong Provincial Key Laboratory of Protein Function and Regulation in Agricultural Organisms, College of Life Sciences, South China Agricultural University, Guangzhou, China; ^2^Guangdong Food and Drug Vocational College, Guangzhou, China

**Keywords:** *Xanthomonas campestris* pv. *campestris*, branched-chain fatty acid biosynthesis, branched-chain amino acids, diffusible signal factor, pathogenesis

## Abstract

In bacteria, the metabolism of branched-chain amino acids (BCAAs) is tightly associated with branched-chain fatty acids (BCFAs) synthetic pathways. Although previous studies have reported on BCFAs biosynthesis, more detailed associations between BCAAs metabolism and BCFAs biosynthesis remain to be addressed. In this study, we deleted the *ilvC* gene, which encodes ketol-acid reductoisomerase in the BCAAs synthetic pathway, from the *Xanthomonas campestris* pv. *campestris* (*Xcc*) genome. We characterized gene functions in BCFAs biosynthesis and production of the diffusible signal factor (DSF) family signals. Disruption of *ilvC* caused *Xcc* to become auxotrophic for valine and isoleucine, and lose the ability to synthesize BCFAs via carbohydrate metabolism. Furthermore, *ilvC* mutant reduced the ability to produce DSF-family signals, especially branched-chain DSF-family signals, which might be the main reason for *Xcc* reduction of pathogenesis toward host plants. In this report, we confirmed that BCFAs do not have major functions in acclimatizing *Xcc* cells to low temperatures.

## Introduction

Bacterial survival depends on the ability to modulate membrane fluidity to acclimatize cells to different environments (Campbell and Cronan, 2001; Lu et al., 2004; Zhang and Rock, 2008). Unsaturated, shorter, and *anteiso*-branched fatty acids all increase membrane fluidity compared with saturated, longer, and *iso*-branched fatty acids (Suutari and Laakso, 1994; Zhang and Rock, 2008). The branched-chain fatty acids (BCFAs), including *iso*-fatty acids and *anteiso*-fatty acids, are common components of the Gram-positive bacterial membrane (Kaneda, 1991). Therefore, BCFAs are important for adaptation to environmental conditions for most Gram-positive bacteria (Zhu et al., 2005a,b; Giotis et al., 2007; Santiago et al., 2012).

**FIGURE 1:**
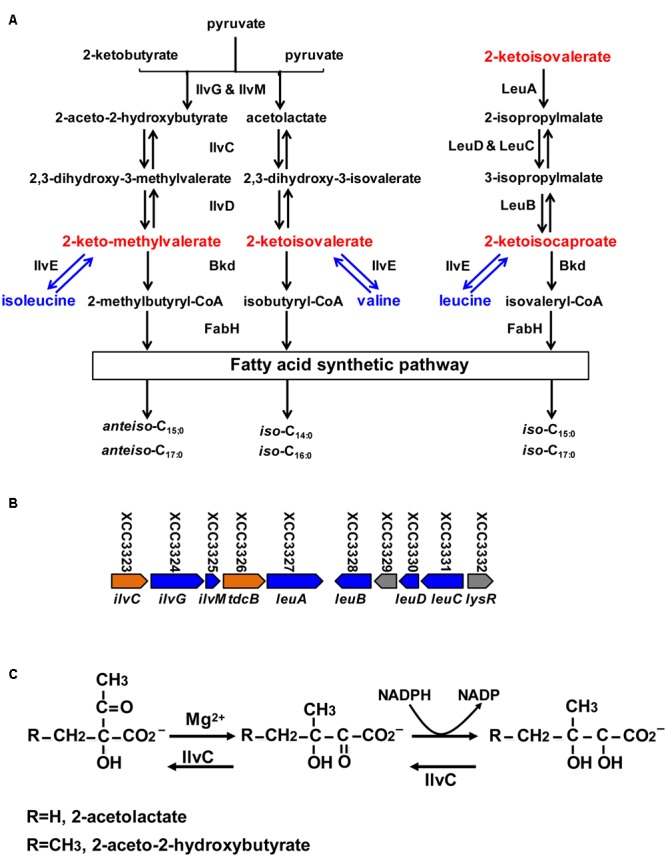
Proposed branched-chain amino acid (BCAA) metabolic pathway, organization of BCAA synthetic gene cluster in *Xcc*, reaction catalyzed by IlvC and alignment of *Xcc* and *Pseudomonas aeruginosa* IlvC. **(A)** Proposed BCAA metabolic pathway in bacteria. IlvGM, acetohydroxy acid synthase; IlvC, keto-acid reductoisomerase; IlvD, dihydroxy-acid dehydratase; IlvE, transaminase; Bkd, branched-chain keto acid dehydrogenase; LeuA, 2-isopropylmalate synthase; LeuCD, isopropylmalate isomerase; LeuB, 3-isopropyl malate dehydrogenase; FabH, 3-ketoacyl-acyl carrier protein synthase III. **(B)** Organization of genes required for BCAA synthesis in *Xcc*. **(C)** Biochemical reaction catalyzed by IlvC.

The biosynthetic mechanism of BCFAs has been well studied in some Gram-positive bacteria such as *Listeria monocytogenes, Staphylococcus aureus*, and *Bacillus subtilis* (Choi et al., 2000; Qiu et al., 2005; Singh et al., 2009). The basic steps in the fatty acid synthesis cycle of these Gram-positive bacteria are similar to *Escherichia coli* (Lu et al., 2004; White et al., 2004; Zhang and Rock, 2008). However, the substrate specificity of FabH in Gram-positive bacteria is distinct from *E. coli*. In *E. coli*, FabH prefers to use acetyl-CoA or propionyl-CoA as primers, leading to production of only straight-chain fatty acids in this organism. The FabH proteins from the Gram-positive bacteria are highly selective in accepting branched-chain acyl-CoAs (including 2-methylbutyryl-CoA, isobutyryl-CoA and isovaleryl-CoA) as primers to initiate fatty acid synthesis and primarily *iso*- and *anteiso*-BCFAs are produced in these bacteria (Choi et al., 2000; Qiu et al., 2005; Singh et al., 2009) (**Figure 1A**).

Branched-chain acyl-CoAs come from oxidative decarboxylation of branched-chain 2-keto acids, including 2-keto-methylvalerate (KMV), 2-ketoisovalerate (KIV) and 2-ketoisocaproate (KIC) catalyzed by the branched-chain 2-keto acid dehydrogenase (Bkd) complex (**Figure 1A**) (Lu et al., 2004). Thus, Bkd is also a critical enzyme in the BCFA synthetic pathway (Lu et al., 2004). Its functions in BCFA synthesis and adaptation to environmental stress in *L. monocytogenes, B. subtilis*, and *S. aureus* have been well studied (Oku and Kaneda, 1988; Wang et al., 1993; Zhu et al., 2005a,b; Singh et al., 2008; Sun and O’Riordan, 2010).

In bacteria, branched-chain 2-keto acids are produced in two ways: from transamination of exogenous branched-chain amino acids (BCAAs) (including isoleucine, valine and leucine), and from carbohydrate metabolism via the *de novo* BCAAs synthetic pathway (**Figure 1A**). Although BCAAs aminotransferases are reported to be involved in BCFAs synthesis in *Lactococcus lactis, S. mutans*, and *S. carnosus* (Santiago et al., 2012), little is known about the physiological functions of *de novo* synthesis of branched-chain 2-keto acids in BCFA synthesis in bacteria.

The general *de novo* synthetic pathway of branched-chain 2-keto acids is described in **Figure 1A**. First, KIV and KMV are synthesized in two parallel pathways through a single set of three enzymes (**Figure 1A**), including acetohydroxy acid synthase (EC 4.1.3.18) (IlvGM), ketol-acid reductoisomerase (EC 1.1.1.86) (IlvC) and dihydroxy-acid dehydratase (EC 4.2.1.9) (IlvD), while KIC is converted from KIV by three enzymes in succession: 2-isopropylmalate synthase (EC 2.3.3.13) (LeuA), isopropylmalate isomerase (EC 4.2.1.33) (LeuDC), and 3-isopropylmalate dehydrogenase (EC 1.1.1.85) (LeuB) (**Figure 1A**).

*Xanthomonas campestris* pv. *campestris* (*Xcc*), the causal agent of crucifer black rot, is possibly the most important disease of crucifers worldwide (He and Zhang, 2008; Ryan and Dow, 2011). *Xcc* is a rod-shaped, aerobic Gram-negative, non-spore-forming bacterium that produces BCFAs, which account for approximately 50% of the total cellular fatty acids (Kaneda, 1991; Yu et al., 2016). In addition, at least three branched-chain diffusible signal factor (DSF)-family signals are known in *Xcc*: DSF (*cis*-11-methyl-2-dodecenoic acid [11-Me-C_12_:Δ^2^]), CDSF (*cis*-11-methyldodeca-2,5-dienoic acid [11-Me-C_12_: Δ^2,5^]), and IDSF (*cis*-10-methyl-2-dodecenoic acid [10-Me-C_12_:Δ^2^]) (Deng et al., 2015; Zhou et al., 2015a,b). These are involved in quorum sensing (QS) mechanisms to regulate the production of a range of virulence determinants, including extracellular polysaccharides (EPS), extracellular enzymes (proteases, pectinases, and cellulases), and a type III secretion system (He and Zhang, 2008; Ryan and Dow, 2011). *Xcc* FabH, like FabHs from Gram-positive bacteria, prefers to use branched-chain acyl-CoAs as primers to initiate BCFAs synthesis (Yu et al., 2016). However, little is known about how *Xcc* produces branch-chain acyl-CoAs. A previous study showed that leucine and valine are the primary precursors for DSF biosynthesis and isoleucine is the primary precursor for IDSF biosynthesis (Zhou et al., 2015b). This finding indicates that BCAAs metabolism is tightly associated with BCFAs synthetic pathways and production of DSF-family signals. However, the metabolism of BCAAs and their relationship with BCFA synthesis in *Xcc* are still unclear.

The *Xcc* genome encodes a complete set of genes required for BCAAs metabolism (Qian et al., 2005; Thieme et al., 2005) (**Figure 1B**). In this report, we focused on ketol-acid reductoisomerase (putatively encoded by XCC3323, *ilvC*), which is the key enzyme in *de novo* synthesis in the BCAA pathway. To study its physiological functions in *Xcc*, we constructed an *ilvC* deletion mutant by homologous recombination. Then, we tested the growth of *ilvC* mutant under various conditions and analyzed the fatty acid composition of the *ilvC* mutant by gas chromatograph-mass spectrometer (GC-MS). We also assayed the production of DSF-family signals by the *ilvC* mutant and its pathogenicity toward host plants.

## Materials and Methods

### Materials

The 2-acetoxyl-2-methyl-ethyl acetoacetate, NADPH, 2-ketoisovalerate (KIV), 2-ketoisocaproate (KIC), 2-keto-methylvalerate (KMV), [^13^C] glucose, and antibiotics were from Sigma–Aldrich (St. Louis, MO, United States). Takara Biotechnology Co. (Dalian, China) provided the molecular biology reagents. Novagen (Madison, WI, United States) provided the pET vectors. Ni-agarose columns were from Invitrogen (Carlsbad, CA, United States). Agilent Technologies (Palo Alto, CA, United States) provided HC-C18 HPLC columns and Bio-Rad (Hercules, CA, United States) provided Quick Start Bradford dye reagent. All other reagents were of the highest available quality. Sangon Biotechnology Co. (Shanghai, China) synthesized oligonucleotide primers.

### Bacterial Strains, Plasmids, and Growth Conditions

Strains and plasmids used in this study are in Supplementary Table S1. *E. coli* strains were grown in Luria-Bertani medium at 37°C. *Xcc* strains were grown at 30°C in media listed in Supplementary Table S3. When required, antibiotics were added at 100 μg/mL sodium ampicillin, 30 μg/mL kanamycin sulfate, 30 μg/mL gentamicin for *E. coli* or 10 μg/mL for *Xcc*, and 50 μg/mL rifampicin. Bacterial growth in liquid medium was determined by measuring optical density at 600 nm (OD_600_) using a Bioscreen-C Automated Growth Curves Analysis System (OY Growth Curves FP-1100-C, Helsinki, Finland).

### Protein Expression and Purification

To clone the *Xcc ilvC* gene, genomic DNA extracted from strain *Xcc* Xc1 was used for PCR amplification with *Pfu* DNA polymerase, using primers in Supplementary Table S2. PCR products were inserted into pET-28b (+) to produce plasmids pKH2. The *ilvC* gene was confirmed by nucleotide sequencing by Shanghai Sangon Inc. (Shanghai, China). *Xcc ilvC* with a vector-encoded His_6_-tagged N-terminus was expressed in *E. coli* BL21 (DE3), and purified with Ni-NTA agarose (Qiagen, Chatsworth, CA, United States) using a nickel-ion affinity column (Qiagen). Protein purity was monitored by SDS-PAGE.

### Spectrophotometric Assays of Ketol-Acid Reductoisomerase Activity

The enzymatic activity assays for IlvC were as described previously (Brinkmann-Chen et al., 2013). Briefly, reaction mixtures contained 250 mmol/L potassium phosphate (pH 7.0), 1 mmol/L DTT, 200 μmol/L NADPH, 10 mmol/L MgCl_2_, and various concentrations of 2-acetolactic produced from hydrolyzation of 2-acetoxyl-2-methyl-ethyl acetoacetate by sodium hydroxide, in a final volume of 100 μL. Reactions were initiated by adding 1 μg IlvC, and ketol-acid reductoisomerase activity of IlvC determined by monitoring the rate of NADPH oxidation at 340 nm using extinction coefficient 6220 M^-1^.

### Deletion of *Xcc ilvC* and Complementation

To disrupt the *Xcc ilvC* gene, the pK18mobsacB-borne in-frame deletion suicide plasmid pKH5 was constructed (Supplementary Figure S2). The 500-bp DNA fragments flanking the *ilvC* gene were amplified with *Pfu* DNA polymerase using *Xcc* genomic DNA as template, and either *Xcc* ilvC EcoRI and *Xcc* ilvC up1 (for Up ilvC), or *Xcc* ilvC down1 and *Xcc* ilvC HindIII (for Dn ilvC), as primers (Supplementary Table S2). Fragments were purified and joined by overlapping PCR. The fused fragment was digested with EcoRI and HindIII, and inserted in pK18mobscaB (Schafer et al., 1994) to obtain plasmid pKH5. Following mating derivatives of *E. coli* strain S17-1 carrying suicide plasmid pKH5 with *Xcc* Xc1 on NYG (Supplementary Table S3) plates for 36 h at 30°C (Supplementary Figure S2), cells were suspended in NYG medium, and appropriate dilutions were inoculated onto NYG plates containing rifampicin to select against the donor strain, plus kanamycin to select for integration of the non-replicating plasmid into the recipient chromosome. A single-crossover integrant colony was spread on NYG medium without kanamycin at 30°C for 36 h, and after appropriate dilutions, culture was spread on NYG plates containing 15% sucrose. Colonies sensitive to kanamycin were screened by PCR using primers in Supplementary Table S2, and *ilvC* deletion strain *Xcc* KH1 was obtained (Supplementary Figures S2B,C). Using a similar approach, we deleted the *ilvC* gene from *Xcc* Xc1 Δ*rpfBC* for the *Xcc* KH2 mutant. For single-copy complementation of the *ilvC* mutant, the coding region of *ilvC* plus 600 bp upstream of the *ilvC* translational start codon was PCR amplified and cloned into a versatile Mini-Tn7 delivery vector (mini-Tn7T-Gm). The resulting plasmid was transferred into strain Xc1 KH1 or Xc1 KH2 by electroporation, with selection as described previously (Jittawuttipoka et al., 2009). The KH3 and KH4 strains were obtained.

### Analysis of Fatty Acid Composition

Bacterial cultures were grown aerobically for 2–4 days. Cells were harvested and washed three times with sterile water. Fatty acid methyl esters were synthesized and extracted as described previously (Stead, 1989). Cellular lipids were saponified by addition of 2 mL sodium hydroxide/methanol solution at 100°C for 40 min with shaking (800 rpm). Fatty acids were methylated by addition of 4 mL hydrochloric acid/methanol solution at 80°C for 30 min, and cooled to below 20°C. Fatty acid methyl esters were obtained by three extractions with 1 mL petroleum ether. Solvent was removed under a stream of nitrogen, and residue dissolved in 100 μL of hexane. The crude extract was filtered with 0.22-μm Mini-star units and 2 μL of extract was analyzed by gas chromatography-mass spectrometry (GC-MS). Samples were analyzed with a GC-MS system (Agilent 5975c) with chromatographic column DB 5MS. The oven temperature was held at 100°C for 5 min, changed at 10°C/min to 200°C and held for 5 min, and changed at 10°C/min to 250°C and held for 5 min. Electron impact ionization (EI^+^, 70 eV) was used for all samples. Mass spectrometry was carried out at 1 sec/scan, m/z 35-500, 1 kV, and data were analyzed by the NIST 08 database.

For analysis of ^13^C-labeled fatty acids, 10 mL cultures of Xc1 or KH1 grown in XOLN medium for 1 day were harvested and washed three times in PBS (Supplementary Table S3). Bacterial cells were re-suspended in 10 mL XO medium (Supplementary Table S3) and 500 μL transferred into 50 mL XO medium supplemented with 1 mg/mL ^13^C-glucose (for Xc1) or 1 mg/mL ^13^C-glucose plus 100 μmol/L valine and isoleucine mixtures (for KH1). Cells were grown at 30°C for 2 days. Fatty acids were extracted and analyzed as described above.

### Detection of DSF Signals Components in *Xcc* Culture Supernatant

The protocol for extraction and purification of DSF family components was described previously (Deng et al., 2015; Zhou et al., 2015a,b). *Xcc* strains were cultured in liquid medium for 36 h and 50 mL bacterial supernatant was collected by centrifugation at 4000 × *g* for 15 min at 4°C. The pH of supernatants was adjusted to 4.0 by adding hydrochloric acid prior to two extractions with an equal volume of ethyl acetate. Ethyl acetate fractions were collected and solvent removed by rotary evaporation to dryness at 40°C. Residue was dissolved in 100 μL methanol. Crude extract was subjected to 0.22-μm Mini-star filtration and filtrate was concentrated to 100 μL. Extract (10 μL) was injected into a C18 reverse-phase HPLC column (4.6 mm × 250 mm, Agilent Technologies), eluted with water in methanol (23:77 v/v, 0.1% formic acid) at a flow rate of 1 mL/minute in a Waters HPLC E2695 system (Waters, Milford, MA, United States) with a UV220 detector.

For detection of DSF signals produced by resting *Xcc* cells, 50 mL KH2 cultures grown in XOLN medium for 24 h were harvested and washed three times using PBS buffer. Cells were re-suspended in 50 mL PBS buffer and grown for 2 h. Cultures were harvested and washed three times using PBS buffer, and transferred to PBS medium supplemented with nutrients, and cells were cultivated at 30°C for 6 h. DSF family signals in supernatants were detected as described above.

### Pathogenicity Tests

Virulence was tested using potted Chinese radishes (*Raphanus sativus* L. var. radiculus Pers.) as described previously (Dow et al., 2003). Bacteria grown overnight in NYG medium were washed and re-suspended in PBS buffer to OD_600_ 0.1. For leaf clippings, the last completely expanded leaf was cut and dipped in bacterial suspensions at OD_600_ 0.1. Lesion length was measured 14 days after inoculation on 30 leaves for each strain tested.

### Statistical Analyses

Analysis of variance for experimental datasets used Graphpad prism 5.0. Significant effects of treatment were determined using *F*-values (*P* = 0.05). When a significant *F* test was obtained, separation of means was accomplished by Fisher’s protected least significant difference at *P* ≤ 0.05.

## Results

### *Xcc* XCC3323 Encodes Ketol-Acid Reductoisomerase (IlvC)

**FIGURE 2:**
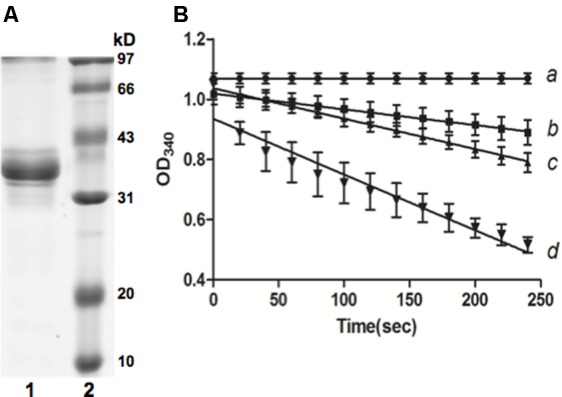
Purification of *Xcc* IlvC from *Escherichia coli* BL21 (DE3) and IlvC activity by reduction of 2-acetolactate. **(A)** Purification of IlvC by native nickel-chelate chromatography. The purified protein was analyzed by 12.5% SDS-PAGE. Lane 1, IlvC protein; lane 2, molecular mass markers. **(B)** Reduction of 2-acetolactate by IlvC. Reaction was followed by NADPH oxidation. a, b, c, and d represents the final concentration of α-acetolactate in reaction mixtures: 0, 0.53, 2.65, and 5.83 mmol/L, respectively.

The genome of *Xcc* strain ATCC 33913 possesses an entire gene cluster required for the biosynthetic pathway of BCAAs (valine, leucine and isoleucine) (**Figure 1B**). In this gene cluster, XCC3323 (*ilvC*) is annotated to encode ketol-acid reductoisomerase (**Figure 1C**). Protein sequence alignments indicated that *Xcc* IlvC shared 56.9% identical residues with *Pseudomonas aeruginosa* IlvC (Eom et al., 2002; Ahn et al., 2003), and conserved regions I–V defined by the X-ray crystallography structure of *P. aeruginosa* IlvC were present in *Xcc* IlvC (Dumas et al., 2001; Tyagi et al., 2005) (Supplementary Figure S1). The residues in ketol-acid reductoisomerase that are involved in binding metal ions, an inhibitor and NADPH were conserved in *Xcc* IlvC (Dumas et al., 2001; Tyagi et al., 2005) (Supplementary Figure S1). To identify if *Xcc ilvC* encoded ketol-acid reductoisomerase, recombinant N-terminal hexahistidine-tagged *Xcc* IlvC was produced. The protein had a monomeric molecular weight of 37 kDa and was purified by nickel chelate chromatography to obtain preparations that gave single bands on SDS-gel electrophoresis (**Figure 2A**). To probe the ketol-acid reductoisomerase activity of *Xcc* IlvC, decreased absorbance of NADPH at 340 nm was monitored spectrophotometrically in reaction mixtures containing 2-acetolactate as a substrate. Along with increased 2-acetolactate concentrations, the rate of NADPH oxidation increased, indicating that *Xcc* IlvC possessed ketol-acid reductoisomerase activity (**Figure 2B**).

### Deletion of *ilvC* Caused *Xcc* Auxotrophy for Valine and Isoleucine

**FIGURE 3:**
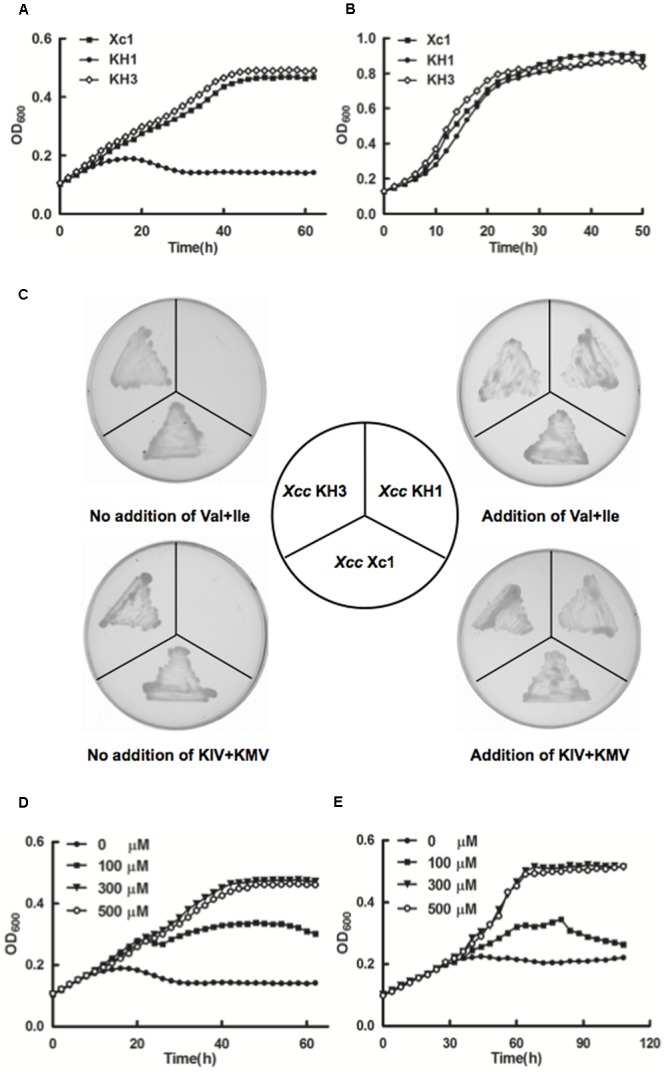
Growth of *Xcc* KH1 mutant. **(A)** Mutant strain *Xcc* KH1 in XOS liquid medium (Supplementary Table S3). Empty diamonds, wild type strain *Xcc* Xc1; filled squares, complementary strain KH3; filled circles, mutant strain KH1. **(B)** Mutant strain *Xcc* KH1 in XOLN liquid medium (Supplementary Table S3). Empty diamonds, wild type *Xcc* Xc1; filled squares, complementary strain KH3; filled circles, mutant strain KH1. **(C)** Mutant strain *Xcc* KH1 on XOS plates supplemented with valine and isoleucine or KIV and KMV mixtures. Val, valine; Ile, isoleucine; KIV, 2-ketoisovalerate; KMV, 2-keto-methylvalerate. **(D,E)** Mutant *Xcc* KH1 in XOS liquid medium supplemented with valine and isoleucine mixtures at 30°C **(D)** or 15°C **(E)**. Filled circles, no supplement; filled squares, 100 μmol/L valine and isoleucine mixture; filled triangles, 300 μmol/L valine and isoleucine mixture; empty circles, 500 μmol/L valine and isoleucine mixture. OD_600_ of cultures was monitored using a Bioscreen-C Automated Growth Curves Analysis System (OY Growth Curves FP-1100-C, Helsinki, Finland). Error bars mean ± standard deviation (*n* = 3). All experiments were repeated three times with similar results.

Ketol-acid reductoisomerase (IlvC) catalyzes the conversion of acetohydroxy acids into dihydroxy valerates and is a key enzyme in the second step of the BCAAs biosynthetic pathway (Dumas et al., 2001; Bastian et al., 2011; Brinkmann-Chen et al., 2013) (**Figure 1A**). To identify the physiological functions of IlvC in *Xcc*, knockout strain KH1, in which the *ilvC* (XCC3323) gene was deleted, was constructed by allelic replacement (Supplementary Figure S2). Growth of the *Xcc* KH1 (Δ*ilvC*) strain was tested in minimal liquid medium XOS (Supplementary Table S3). The *Xcc* KH1 strain failed to grow in XOS medium, but the complementary strain KH3, in which KH1 carried a single copy of wild type *ilvC* at the *glmS* site, had fully restored growth in XOS medium (**Figure 3A**). However, in XOLN medium (Supplementary Table S3), *Xcc* KH1 grew as well as the wild type strain *Xcc* Xc1 (**Figure 3B**). These results indicated that deletion of *ilvC* caused *Xcc* to fail to produce some nutrients from carbohydrate metabolic pathways to support *Xcc* growth. Given IlvC is the key enzyme of the BCAA biosynthetic pathway, we tested the growth of strain *Xcc* KH1 in XOS medium supplemented with valine, leucine, or isoleucine. None of the single BCAAs was able to restore *Xcc* KH1 growth on XOS plates (data not shown). However, supplementing of XOS medium with 100 μmol/L mixtures of valine and isoleucine restored *Xcc* KH1 strain growth on XOS plates (**Figure 3C**). While the same concentration of valine and leucine or leucine and isoleucine mixtures did not restore strain *Xcc* KH1 growth. These results suggested that deletion of *ilvC* caused *Xcc* to fail to produce valine and isoleucine. Generally, in cells, production of BCAAs comes from transamination of branched-chain 2-keto acids KIV, KIC and KMV, respectively. Thus, to determine if deletion of *ilvC* led *Xcc* to fail to produce branched-chain 2-keto acids from the carbohydrate metabolic pathway, we supplemented XOS medium with KIV, KIC, and KMV and tested if strain *Xcc* KH1 grew. *Xcc* KH1 grew as well as the wild type strain *Xcc* Xc1 when XOS medium was supplemented with KIV and KMV mixtures (**Figure 3C**). However, single branched-chain 2-keto acids, or KIV and KIC, or KIC and KMV mixtures did not restore strain KH1 growth (data not shown). This result confirmed that *Xcc ilvC* encoded ketol-acid reductoisomerase and deletion of *ilvC* caused *Xcc* to fail to produce KIV and KMV from carbohydrate metabolism, and caused *Xcc* auxotrophy for valine and isoleucine.

We analyzed mutant strain *Xcc* KH1 reaction to addition of various concentrations of valine and isoleucine mixtures. Addition of 100 μmol/L mixtures supported *Xcc* KH1 growth, although growth was weaker than the wild type strain Xc1 in XOS medium. However, when mixtures were increased to 300 μmol/L, *Xcc* KH1 growth reached the level of wild strain Xc1 growth in XOS medium. Addition of 500 μmol/L mixtures did not improve growth of mutant strain *Xcc* KH1 (**Figure 3D**). The effects of KIV and KMV mixtures on *Xcc* KH1 growth were tested and similar results were obtained: 300 μmol/L KIV and KMV mixtures best supported *Xcc* KH1 growth (Supplementary Figure S3A).

The effects of various concentrations of valine and isoleucine mixtures on *Xcc* KH1 growth at low temperature were also determined. Higher concentrations of valine and isoleucine mixtures did not increase *Xcc* KH1 growth at low temperature compared with growth of the wild type strain Xc1 in XOS medium at the same temperature (**Figure 3E**). Higher concentrations of KIV and KMV mixtures did not increase *Xcc* KH1 growth at low temperature either (Supplementary Figure S3B).

### Deletion of *ilvC* Resulted in *Xcc* Not Synthesizing BCFAs *de Novo* from the Carbohydrate Metabolic Pathway

Deletion of *ilvC* caused *Xcc* auxotrophy for valine and isoleucine. Therefore, we hypothesized that the fatty acid profile of mutant strain *Xcc* KH1, especially for BCFAs, would be distinct from wild type strain Xc1. We analyzed the fatty acid composition of total lipid extracts from *Xcc* KH1 grown in XOLN medium at 30°C by GC-MS. The species and amounts of fatty acids of *Xcc* KH1, especially BCFAs, were almost the same as that in *Xcc* Xc1 (**Table 1**). To test if temperature affected the fatty acid profile of strain *Xcc* KH1, the fatty acid composition of *Xcc* KH1 grown at 15°C was determined. The fatty acid profile of *Xcc* KH1 did not differ from *Xcc* Xc1 under this temperature condition (**Table 1**). These results suggested that deletion of *ilvC* did not affect BCFA synthesis in strain *Xcc*.

**Table 1:** Fatty acid composition of total lipid extracts from *Xcc* Xc1 and *ilvC* mutant strains grown in XOLN medium^a^.

Fatty acids (%)	*Xcc* Xc1		*Xcc* KH1		*Xcc* KH3	
	30°C	15°C	30°C	15°C	30°C	15°C
n-C_14:0_ 3-OH^b^	3.51 ± 0.53	6.34 ± 0.70	4.06 ± 0.60	6.49 ± 0.10	3.49 ± 0.17	6.68 ± 0.43
*iso*-C_15:0_	15.56 ± 0.84	8.78 ± 0.44	16.39 ± 1.12	9.39 ± 0.11	10.33 ± 0.11	7.96 ± 0.12
*anteiso-*C_15:0_	17.42 ± 0.92	11.88 ± 0.34	16.47 ± 0.74	13.01 ± 0.32	14.12 ± 0.25	10.21 ± 0.06
n-C_15:0_	2.67 ± 0.90	1.98 ± 0.23	1.69 ± 0.17	1.64 ± 0.39	1.80 ± 0.31	0.34 ± 0.04
*iso*-C_16:0_	5.44 ± 0.03	2.57 ± 0.13	4.79 ± 0.04	2.48 ± 0.18	5.04 ± 0.09	2.23 ± 0.03
n-C_16:1_ *cis*-9	15.11 ± 0.31	32.30 ± 0.51	16.69 ± 0.07	30.09 ± 0.29	18.05 ± 1.21	33.01 ± 1.08
n-C_16:0_	9.13 ± 0.87	14.29 ± 0.50	10.19 ± 1.09	12.47 ± 0.11	12.06 ± 0.73	15.84 ± 0.22
n-C_17:1_ *cis*-9	11.57 ± 0.58	7.27 ± 0.94	11.23 ± 0.37	8.03 ± 1.08	12.00 ± 0.75	7.90 ± 0.36
*iso*-C_17:0_	9.88 ± 0.09	5.20 ± 0.30	9.28 ± 0.37	5.87 ± 0.49	10.76 ± 0.05	6.05 ± 0.24
*anteiso*-C_17:0_	2.41 ± 0.09	0.99 ± 0.11	1.92 ± 0.20	1.48 ± 0.17	3.08 ± 0.50	1.70 ± 0.02
n-C_17:1_ *cis*-10	1.96 ± 0.97	2.64 ± 0.23	2.38 ± 0.06	2.96 ± 0.12	1.80 ± 0.34	1.14 ± 0.17
n-C_18:1_ *cis*-11	4.46 ± 0.17	5.31 ± 1.08	4.27 ± 0.62	5.44 ± 0.58	6.65 ± 0.20	6.42 ± 0.31
n-C_18:0_	0.88 ± 0.26	0.47 ± 0.06	0.65 ± 0.12	0.65 ± 0.16	0.85 ± 0.17	0.61 ± 0.04
Total UFAs	33.10 ± 0.63	47.51 ± 2.15	34.57 ± 0.91	46.52 ± 1.11	38.50 ± 0.77	48.47 ± 0.88
Total BCFAs	50.71 ± 1.67	29.42 ± 1.05	48.85 ± 1.25	32.23 ± 1.12	43.33 ± 0.82	28.16 ± 0.29
*iso*-BCFAs	30.88 ± 0.87	16.55 ± 0.72	30.46 ± 0.71	17.74 ± 0.69	26.13 ± 0.21	16.24 ± 0.29
*anteiso*-BCFAs	19.83 ± 0.83	12.87 ± 0.33	18.39 ± 0.55	14.49 ± 0.45	17.20 ± 0.68	11.91 ± 0.05
*anteiso*-/*iso*-	0.64 ± 0.01	0.78 ± 0.01	0.60 ± 0.01	0.82 ± 0.01	0.66 ± 0.02	0.73 ± 0.01

*^a^Cells were grown in XOLN medium for 36 h at 30°C or 15°C. Total lipids were extracted and transesterified to fatty acid methyl esters, and products identified by GC-MS. Values are percentages of total fatty acids and are the mean ± standard deviations of three independent experiments.*

*^b^n-C_*14:0*_ 3-OH, 3-hydroxyltetradecanoic; *iso*-C_*15:0*_, 13-methyl-tetradecanoic acid; *anteiso*-C_*15:0*_, 12-methyl-tetradecanoic acid; n-C_*15:0*_, pentadecanoic acid; *iso*-C_*16:0*_, 14-methyl-pentadecanoic acid; n-C_*16:1*_*cis*-9, *cis*-9-hexadecenoic acid; n-C_*16:0*_, hexadecanoic acid; *iso*-C_*17:1*_*cis*-9, *cis*-9-15-methyl-hexadecenoic acid; *iso*-C_*17:0*_, 15-methyl-hexadecanoic acid; *anteiso*-C_*17:0*_, 14-methyl-hexadecanoic acid; n-C_*17:1*_*cis*-10, *cis*-10-hexadecenoic acid; n-C_*18:0*_, octadecanoic acid. UFA, unsaturated fatty acid; BCFA, branched-chain fatty acid.*

**FIGURE 4:**
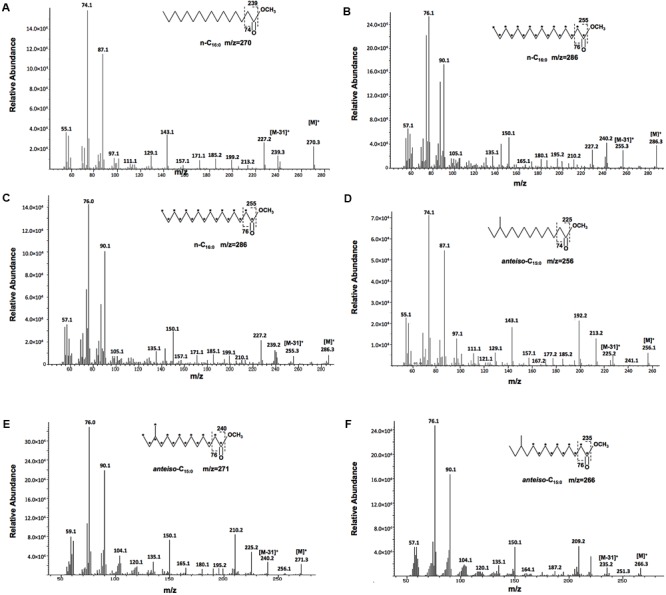
Mass spectra of ^13^C-labeled FAMES. **(A)** Mass spectra of unlabeled methyl hexadecanoate (n-C_16:0_). **(B)** Mass spectra of ^13^C-labeled methyl hexadecanoate from wild type *Xcc* Xc1. **(C)** Mass spectra of ^13^C-labeled methyl hexadecanoate from mutant KH1. **(D)** Mass spectra of unlabeled methyl *anteiso*-pentadecanoate (*anteiso*-C_15:0_). **(E)** Mass spectra of ^13^C-labeled methyl *anteiso*-pentadecanoate from wild type *Xcc* Xc1. **(F)** Mass spectra of ^13^C-labeled methyl *anteiso*-pentadecanoate from mutant KH1. Parent molecular ions ([M^+^]) and select fragment ions are indicated, including for the McLafferty ion.

Then, we determined why deletion of *Xcc ilvC* did not lead to changes in *Xcc* BCFAs composition with an isotope-labeling method. We fed the wild type strain *Xcc* Xc1 and mutant strain *Xcc* KH1 with [^13^C] glucose, which as all six carbons involved in ^13^C labeling, and we analyzed the fatty acid composition as fatty acid methyl esters (FAME) using GC-MS. The fatty acid profiles of the two strains obtained were almost same as the wild type strain fed unlabeled glucose (data not shown). Next, the structure of each ^13^C-labeled FAME peak was assigned using mass spectrometry. Compared to the corresponding unlabeled FAME peak, all ^13^C-labeled FAME molecules from both strains were heavier. For the same species n-C_m:0_ straight-chain fatty acids ester, the m/z of ^13^C-labeled FAME was the same for the wild type and mutant strain, and was m Da heavier than unlabeled FAME. For example, the m/z of unlabeled n-C_16:0_ methyl ester was 270 (**Figure 4A**), and the m/z of ^13^C-labeled n-C_16:0_ methyl ester in both strains was 286 (**Figures 4B,C**). This measurement was 16 Da higher than the unlabeled n-C_16:0_ methyl ester, which indicated that all carbons in n-C_16:0_ fatty acid in both the wild type and mutant strains were from catabolism of ^13^C-labeled glucose. However, for BCFAs, the m/z of ^13^C-labeled FAME from wild type strain Xc1 differed from mutant strain KH1. The m/z of ^13^C-labeled *anteiso*-C_15:0_ methyl ester in the wild type strain was 271 (**Figure 4E**), 15 Da higher than the unlabeled *anteiso*-C_15:0_ methyl ester (**Figure 4D**), indicating that all 15 carbons of *anteiso*-C_15:0_ in wild type strain Xc1 were labeled by ^13^C. While the m/z of ^13^C-labeled *anteiso*-C_15:0_ methyl ester in mutant strain KH1 was 266 (**Figure 4F**), which was 10 Da higher than unlabeled *anteiso*-C_15:0_ methyl ester and 5 Da less than the ^13^C-labeled *anteiso*-C_15:0_ methyl ester in the wild type strain. These results suggested that only 10 carbons in *anteiso*-C_15:0_ in mutant strain KH1 were from the catabolism of ^13^C-labeled glucose, while five unlabeled carbons came from the exogenous isoleucine. We also analyzed the ^13^C-labeled *iso*-C_16:0_ methyl ester in the mutant strain, and concluded that four unlabeled carbons in the *iso*-C_16:0_ methyl ester of the mutant strain came from exogenous supplement with valine (Supplementary Figure S4). All these results confirmed that deletion of *ilvC* resulted in *Xcc* not synthesizing BCFAs *de novo* from the carbohydrate metabolic pathway and the mutant strain used exogenous BCAAs to produce BCFAs and support strain growth.

### *Xcc* Did Not Mainly Rely on Modulation of BCFAs to Respond to Low Temperature

To further investigate use of exogenous BCAAs to produce BCFAs by the *ilvC* deletion mutant, different concentrations of valine and isoleucine mixtures were added to XOS medium and the fatty acid composition of strain KH1 grown at 30°C or 15°C was determined by GC-MS. Compared to the fatty acid composition of wild type strain Xc1 grown in XOS medium at 30°C, supplement with valine and isoleucine mixtures increased the amount of BCFAs in mutant strain KH1, especially the amount of *anteiso*-C_15:0_ fatty acid (**Table 2**). As the concentrations of valine and isoleucine mixtures increased, the total BCFAs from mutant strain KH1 also increased (**Table 2**). Under these conditions, the total unsaturated fatty acids (UFAs) of strain KH1 were decreased. This result suggested that to a certain degree, BCFAs, especially *anteiso*-C_15:0_, replaced the function of UFAs to maintain membrane fluidity in *Xcc*. However, although supplementing XOS medium with valine and isoleucine mixtures also increased BCFAs in strain KH1 grown at 15°C, BCFAs were not enhanced in strain KH1 with higher concentrations of valine and isoleucine supplements (**Table 2**). This result indicated that *Xcc* did not mainly rely on modulation of BCFAs to respond to low temperature. To confirm this finding, we determined the fatty acid composition of the wild type strain grown at 15°C in XOS medium supplemented with valine and isoleucine mixture and obtained similar results (Supplementary Table S4) that supplementing with higher concentrations of valine and isoleucine mixtures did not significantly increase BCFAs in *Xcc* cells at low temperature.

**Table 2:** Fatty acid composition of total lipid extracts from *Xcc* Xc1 and *ilvC* mutant strains grown in XOS medium supplemented with valine and isoleucine^a^.

Fatty acids (%)	Xc1		KH1 V+I (100 μmol/L)		KH1 V+I (300 μmol/L)	
	30°C	15°C	30°C	15°C	30°C	15°C
n-C_14:0_ 3-OH^b^	2.66 ± 1.43	3.01 ± 2.81	8.06 ± 3.32	6.16 ± 0.44	6.22 ± 0.35	5.03 ± 0.63
*iso*-C_15:0_	6.30 ± 0.61	5.64 ± 0.49	3.16 ± 0.86	2.12 ± 0.11	4.09 ± 0.39	2.02 ± 0.48
*anteiso-*C_15:0_	8.50 ± 0.51	7.50 ± 0.22	19.01 ± 2.92	22.53 ± 0.00	25.50 ± 1.75	20.33 ± 1.59
n-C_15:0_	0.58 ± 0.18	0.18 ± 0.31	0.21 ± 0.37	0.71 ± 0.08	0.97 ± 0.15	0.70 ± 0.61
*iso*-C_16:0_	3.67 ± 0.37	3.74 ± 0.13	2.33 ± 0.15	1.80 ± 0.19	4.38 ± 0.23	2.70 ± 0.67
n-C_16:1_ *cis*-9	19.16 ± 2.15	33.68 ± 3.47	16.78 ± 0.75	25.22 ± 0.87	13.77 ± 0.92	25.01 ± 1.66
n-C_16:0_	21.05 ± 0.69	19.91 ± 2.56	18.58 ± 0.56	13.94 ± 1.27	12.79 ± 0.25	13.82 ± 0.90
*iso*-C_17:1_ *cis*-9	13.21 ± 0.61	8.01 ± 5.63	8.85 ± 1.21	5.24 ± 0.11	8.31 ± 1.49	8.62 ± 1.00
*iso*-C_17:0_	4.58 ± 0.40	3.90 ± 0.13	2.52 ± 0.13	2.37 ± 0.08	4.00 ± 0.32	2.83 ± 0.18
*anteiso*-C_17:0_	0.90 ± 0.89	0.35 ± 0.61	4.87 ± 1.23	5.65 ± 0.27	6.50 ± 0.51	4.90 ± 0.30
n-C_17:1_ *cis*-10	0.46 ± 0.8	0.41 ± 0.72	0.37 ± 0.64	2.06 ± 0.11	1.85 ± 0.25	2.07 ± 0.28
n-C_18:1_ *cis*-11	14.19 ± 2.83	10.99 ± 1.36	9.91 ± 3.52	9.13 ± 1.87	8.82 ± 2.76	9.20 ± 0.54
n-C_18:0_	4.71 ± 0.11	2.69 ± 0.41	5.34 ± 2.59	3.1 ± 0.18	2.81 ± 0.10	2.76 ± 0.37
Total UFAs	47.03 ± 2.44	53.09 ± 2.25	35.91 ± 3.68	41.65 ± 1.00	32.75 ± 3.49	44.91 ± 0.37
Total BCFAs	23.96 ± 2.43	21.13 ± 0.80	31.90 ± 2.86	34.46 ± 0.04	44.47 ± 3.08	32.78 ± 1.18
*iso*-BCFAs	14.55 ± 1.31	13.28 ± 0.24	8.01 ± 0.86	6.28 ± 0.23	12.47 ± 0.93	7.55 ± 0.93
*anteiso*-BCFAs	9.40 ± 1.20	7.85 ± 0.66	23.89 ± 2.13	28.18 ± 0.27	32.00 ± 2.19	25.23 ± 1.83
*anteiso*-/*iso*-	0.65 ± 0.04	0.59 ± 0.05	2.99 ± 0.18	4.49 ± 0.20	2.57 ± 0.06	3.40 ± 0.64

*^a^Cells were grown in XOS medium supplemented with valine and isoleucine for 36 h at 30°C or 15°C. Total lipids were extracted and transesterified to fatty acid methyl esters, and products identified by GC-MS. Values are percentages of total fatty acids and are means ± standard deviations of three independent experiments.*

*^b^n-C_*14:0*_ 3-OH, 3-hydroxyltetradecanoic; *iso*-C_*15:0*_, 13-methyl-tetradecanoic acid; *anteiso*-C_*15:0*_, 12-methyl-tetradecanoic acid; n-C_*15:0*_, pentadecanoic acid; *iso*-C_*16:0*_, 14-methyl-pentadecanoic acid; n-C_*16:1*_, *cis*-9-hexadecenoic acid; n-C_*16:0*_, hexadecanoic acid; *iso*-C_*17:1*_*cis*-9, *cis*-9-15-methyl-hexadecenoic acid; *iso*-C_*17:0*_, 15-methyl-hexadecanoic acid; *anteiso*-C_*17:0*_, 14-methyl-hexadecanoic acid; n-C_*17:1*_*cis*-10, *cis*-10-hexadecenoic acid; n-C_*18:1*_*cis*-11, *cis*-11-octadecenoic acid; n-C_*18:0*_, octadecanoic acid. UFA, unsaturated fatty acid; BCFA. branched-chain fatty acid.*

### Deletion of *ilvC* reduced *Xcc* Pathogenesis in Host Plants

**FIGURE 5:**
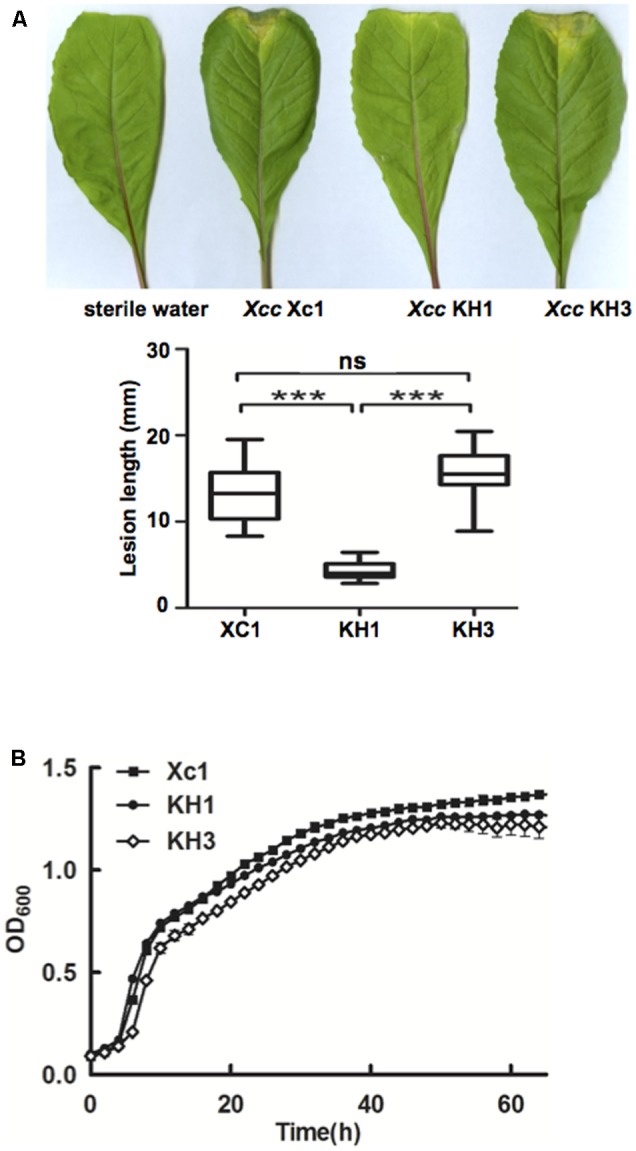
Effects of *ilvC* deletion on *Xcc* virulence. **(A)** Pathogenicity on Chinese radishes of *Xcc* wild type Xc1 and mutant KH1. Virulence of *Xcc* strains was tested by measuring lesion length after introducing bacteria into the Chinese radish “Manjianghong” vascular system by leaf clipping. Values are mean and standard deviation of triplicate measurements, each of 15 leaves. Error bars, mean ± standard deviation. ^∗∗∗^Significant differences (*P* < 0.001, assessed with one-way ANOVA). **(B)** Growth of *Xcc* mutant KH1 in Chinese cabbage extracts (Supplementary Table S3). OD_600_ was monitored using the Bioscreen-C Automated Growth Curves Analysis System (OY Growth Curves FP-1100-C). Filled diamonds, strain Xc1; filled circles, strain KH4; empty diamonds, strain KH2.

*Xcc* is the causal agent of black rot disease of cruciferous vegetables (He and Zhang, 2008). To investigate the effects of *ilvC* deletion on virulence toward host plants, the pathogenesis of mutant strain KH1 was tested. Leaf-clipping virulence assays using Chinese radishes were conducted. The average lesion length caused by wild type strain Xc1 on a Chinese radish leaf was 13.1 mm at 2 weeks after inoculation (**Figure 5A**). Deletion of *ilvC* in strain KH1 significantly reduced average lesion length (4.5 mm) (**Figure 5A**), but complementation with a single copy of wild type *ilvC* fully restored *Xcc* KH1 virulence against host plants; the average lesion length with the complementary strain was 14.5 mm (**Figure 5A**). This result indicated that deletion of *ilvC* led *Xcc* to have reduced virulence against host plants. Deletion of *ilvC* prevents *Xcc* from *de novo* synthesis of BCAAs. Thus, reduced virulence of mutant strain KH1 might have been due to weak growth of host plants caused by *ilvC* deletion. To test this possibility, the growth of strain KH1 in Chinese cabbage extracts was determined. Under these conditions, the growth pattern of mutant strain KH1 was similar to the wild type strain Xc1 (**Figure 5B**), suggesting that *ilvC* deletion did not weaken growth of *Xcc* in host plants significantly, and host plants had enough BCAAs to support mutant growth. Next, we evaluated several pathogenicity-related virulence factors produced by the mutant strain. The activity of extracellular enzymes cellulase, amylase, and protease, and the amount of EPS produced by mutant strain KH1 were not significantly different from the wild type strain Xc1 (data not shown). These results indicated that reduction of *Xcc* pathogenesis by deletion of *ilvC* was not caused by reduction of these pathogenicity-related virulence factors. We also tested the production of DSF-family signals by mutant strain KH1 grown in NYG medium using the biosensor strain. The data showed that during the KH1 growth, the amount of DSF-family signals was lower than that produced by wild type strain Xc1 grown in the same medium (data not shown). However, whether reduction of *Xcc* virulence was due to low-level DSF family signals produced in the *ilvC* mutant needs to be further investigated.

### Deletion of *ilvC* Decreased *Xcc* to Produce Branched-Chain DSF Signals

**FIGURE 6:**
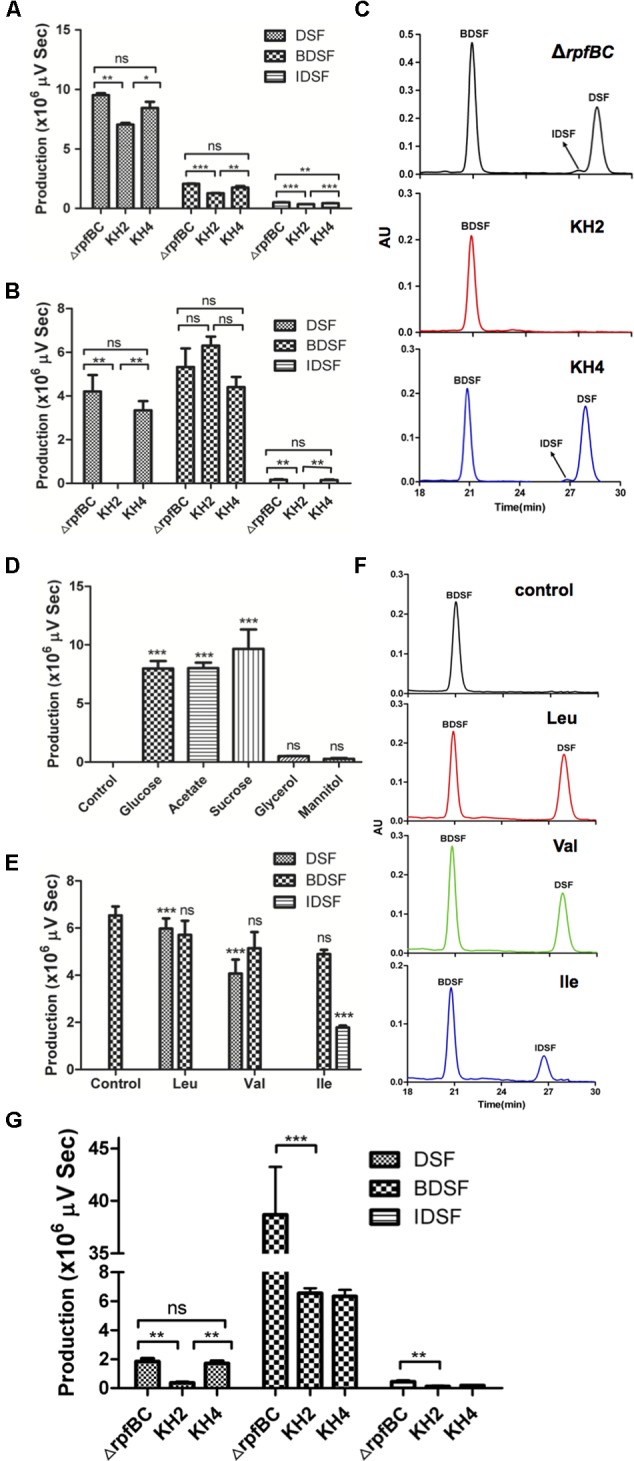
Diffusible signal factor (DSF)-family signals by *ilvC* deletion mutant. **(A)** DSF signals produced by *Xcc ilvC* deletion mutant KH2 grown in NA medium (Supplementary Table S3). Supernatants of 5 mL strain KH2 grown in NA medium for 36 h were collected and DSF signals detected. **(B,C)** DSF signals produced by resting cells of KH2 in PBSS medium (Supplementary Table S3). **(D)** DSF signals produced by resting cells of KH2 in PBS medium (Supplementary Table S3) supplemented with indicated carbon sources. Control, DSF signals produced by resting cells of KH2 in PBS medium (Supplementary Table S3) without carbon source. **(E,F)** DSF signals produced by resting cells of KH2 in PBSS medium supplemented with BCAAs. Control, DSF signals produced by resting cells of KH2 in PBSS medium (Supplementary Table S3) without BCAAs. **(G)** DSF signals produced by *Xcc ilvC* deletion mutant KH2 grown in Chinese cabbage extracts. Supernatants of 50 mL strain KH2 grown for 36 h were collected and DSF signals detected. Error bars, mean ± standard deviation (*n* = 3). ^∗^*P* < 0.05, ^∗∗^*P* < 0.01, ^∗∗∗^*P* < 0.001, assessed with one-way ANOVA. All experiments were repeated three times with similar results. Relative amounts of signal molecules were calculated based on peak areas.

As the Xc1 *rpfBC* double-deletion strain produced higher levels of DSF family signals (Zhou et al., 2015a), to obtain enough DSF family signals for analysis, we constructed mutant strain KH2 by deleting of *ilvC* from the parent strain Xc1 Δ*rpfBC* mutant, and the complementary strain KH4, in which a single copy of wild type *ilvC* was inserted into the *glmS* site of strain KH2 by mini-Tn7 delivery vector. Production of DSF-family signals of the mutant strain KH2 grown in NA medium (Supplementary Table S3) was first tested. All strains produced DSF-family signals: DSF, BDSF, and IDSF, but DSF was the main DSF-family signal under these conditions (**Figure 6A**), consistent with a previous report (Zhou et al., 2015b). However, the amount of each species of DSF-family signals produced by mutant strain KH2 was significantly lower than the Xc1 Δ*rpfBC* strain (*P* < 0.01). The complementary strain KH4 yielded DSF-family signals at the level of the Xc1 Δ*rpfBC* strain. We also examined DSF-family signal production of the mutant strain grown in XLON medium and obtained similar results (Supplementary Figure S5A). These findings indicated that *ilvC* deletion reduced *Xcc* synthesis of DSF-family signals.

Given that *ilvC* deletion caused *Xcc* auxotrophy for valine and isoleucine, we hypothesized it would prevent *Xcc* from producing branched-chain DSF-family signals from carbohydrate metabolism, and branched-chain DSF-family signals from mutant strain KH2 would come from conversion of exogenous BCAAs. To test this hypothesis, we determined DSF-family signals produced by resting cells of mutant strain KH2 in PBSS (Supplementary Table S3). Under these conditions, strain Xc1 Δ*rpfBC* produced three kinds of DSF-family signals: DSF, BDSF, and IDSF. While mutant strain KH2 produced only the BDSF signal. DSF and IDSF, both branched-chain DSF-family signals, were not detected in cultures of strain KH2 (**Figures 6B,C**). The complementary strain KH4 produced three kinds of DSF-family signals at the level of strain Xc1 Δ*rpfBC* (**Figures 6B,C**). To determine if other carbon sources promoted KH2 resting cells to synthesize branched-chain DSF-family signals, we supplemented PBS (Supplementary Table S3) medium with sodium acetate, glucose, mannitol, and glycerol, and DSF-family signal production profiles were determined. None of these candidates promoted KH2 production of branched-chain DSF-family signals (**Figure 6D**). These results indicated that strain KH2 failed to synthesize branch-chain DSF-family signals from carbohydrate metabolism. To determine if branched-chain DSF-family signals produced by mutant strain KH2 were from conversion of exogenous BCAAs, we tested DSF-family signal production by resting cells of mutant strain KH2 in PBSS medium supplemented with valine, leucine or isoleucine. Resting KH2 cells produced DSF when valine or leucine was added to PBSS medium, while addition of isoleucine to PBSS medium resulted in IDSF production (**Figures 6E,F**). We also tested DSF-family signal production by KH2 resting cells when KIV, KIC, or KMV were added to PBSS medium (Supplementary Figures S5B,C). Addition of KIV and KIC resulted in KH2 production of DSF, while addition of KMV resulted in production of the IDSF signal. These results confirmed that the branched-chain DSF-family signals produced by KH2 were from conversion of exogenous BCAAs and that KIV and KIC were the precursors of the DSF signal, and KMV was the precursor of the IDSF signal.

To determine why *ilvC* deletion reduced *Xcc* pathogenesis against host plants, we determined DSF-family signal production by the KH2 strain grown in Chinese cabbage extracts. All strains (Xc1 Δ*rpfBC*, KH2 and KH4) mainly produced DSF and BDSF signals. However, the amount of the DSF-family signals produced by strain KH2 was less than the Xc1 Δ*rpfBC* strain. Although the amount of DSF produced by complementary strain KH4 was almost the same as the amount produced by strain Xc1 Δ*rpfBC*, the KH4 strain did not produce BDSF at the level of the Xc1 Δ*rpfBC* strain (**Figure 6G**). Given that the complementary strain restored virulence against host plants, we hypothesized that the DSF signal was critical for *Xcc* infection of host plants, even though *Xcc* produces more BDSF signal under this condition.

## Discussion

The *ilvC* (XCC3323) gene encodes ketol-acid reductoisomerase, the key enzyme in the biosynthetic pathway for BCAAs in *Xcc*. Our studies confirmed that *ilvC* deletion prevented *Xcc* from using intermediates from carbohydrate metabolism to produce KIV and KMV. KIV is the common precursor for valine and leucine synthesis, while KMV is the precursor for isoleucine (**Figure 1A**). Thus, although *Xcc ilvC* deletion mutant was auxotrophic for BCAAs, only supplementing with valine and isoleucine mixtures or KIV and KMV mixtures restored growth of the *Xcc ilvC* deletion mutant. This result was consistent with studies on ketol-acid reductoisomerase in other bacteria (Aguilar and Grasso, 1991).

Branched-chain acyl-CoAs are primary precursors for synthesis of BCFAs, which come from oxidative decarboxylation of branched-chain 2-keto-acids catalyzed by the Bkd complex (Lu et al., 2004). The *Xcc* genome has a Bkd encoded gene cluster, XCC0427-XCC0430 (Qian et al., 2005; Thieme et al., 2005), which ensures that branched-chain 2-keto-acids convert to branched-chain acyl-CoAs. However, although deletion of *ilvC* prevented *Xcc* producing branched-chain 2-keto-acids from carbohydrate metabolism, the *Xcc ilvC* deletion mutant synthesized BCFAs normally when it grew. This implies that *Xcc* might possess the ability to take up exogenous BCAAs and convert them to branched-chain 2-keto-acids. Indeed, *Xcc* has a BCAA transaminase gene (*ilvE*) in its genome (Qian et al., 2005; Thieme et al., 2005), which converts exogenous BCAAs to branched-chain 2-keto-acids.

Although supplementing with valine and isoleucine mixtures increased the percentage of BCFAs, especially *anteiso*-C_15:0_, and decreased the amount of UFAs in *Xcc* membrane lipids, *Xcc* significantly increased UFAs at low temperature, even when supplemented with high concentrations of valine and isoleucine mixtures at low temperature, which indicated that BCFAs did not have a major effect on response to a decrease in ambient temperature. These results were consistent with previous studies that replacement of *Xcc fabH* with *E. coli fabH* caused *Xcc* to lose the ability to synthesize BCFAs, but the mutant strain grew well at low temperature (Yu et al., 2016). This confirms that the adaptive mechanism to low temperature in *Xcc* differs from that in *L. monocytogenes*, which mainly stimulates *anteiso*-BCFA biosynthesis to increase membrane disorder in response to low temperatures (Zhu et al., 2005a,b).

Deletion of *ilvC* reduced the virulence of *Xcc* against the host plant. Previous studies reported that some *Sinorhizobium meliloti* mutants in genes involved in BCAA biosynthesis are unable to induce nodule formation in host plants (Aguilar and Grasso, 1991). de las Nieves Peltzer et al. (2008) confirmed that auxotrophy for BCAAs causes the nodulation defect of mutants. However, the growth pattern of the *ilvC* mutant strain was similar to the wild type strain in plant extracts, implying that lack of BCAA supplement was not the main cause of the virulence defect of *Xcc ilvC* mutant. The *ilvC* mutant had reduced production of DSF-family signals. Thus, we hypothesized that the reduction of DSF-family signals was the main reason that *Xcc* had weakened virulence against host plants. However, a more detailed mechanism requires further investigation.

The precursors of the DSF-family signal come from the fatty acid synthesis pathway (Zhou et al., 2015b; Yu et al., 2016). However, though *ilvC* deletion mutant produced fatty acid normally, the total amount of DSF-family signal production was reduced during its growth. For *Xcc*, DSF-family signals are secondary metabolic products (He and Zhang, 2008), reaching a maximum during the stationary phase (Wang et al., 2004), while fatty acids are primary metabolites, which are the component of membrane lipids (Zhang and Rock, 2008). Therefore, the BCAAs absorbed from the external environment by the *ilvC* deletion mutant, might be first used to synthesize fatty acids to support *Xcc* cell growth, and then used to produce DSF-family signals. In other words, it might be the difference in distribution of the intermediate products between primary and secondary metabolism that causes *Xcc* to reduce DSF family signal production.

Supplementing PBSS medium with BCAAs or branched-chain 2-keto-acids resulted in resting KH2 strain cells producing different branched-chain DSF-family signals. Adding valine and leucine, or KIV and KIC, led resting cells to produce DSF signals, while adding isoleucine or KMV increased resting cell yield of the IDSF signal. These results were consistent with previous studies (Zhou et al., 2015b), and demonstrated that KIC was the precursor for the DSF signal, and KMV was the precursor for the IDSF signal. However, the amount of IDSF signal produced by strain KH2 in PBSS medium containing isoleucine was lower than the DSF signal produced in PBSS medium supplemented with the same concentrations of valine or leucine. Other researchers also observed that the amount of IDSF is always lower than that of DSF or BDSF at various growth conditions (Deng et al., 2015; Zhou et al., 2015b). Both DSF and IDSF signal are known to come from the dehydration and thiolysis of branch-chain 3-hydroxyacyl-ACPs catalyzed by RpfF (Bi et al., 2012; Zhou et al., 2015b). Our results showed that the addition of valine and isoleucine mixtures could increase the amount of *anteiso*-C_15:0_ in *Xcc* membrane lipids, which suggested that under these conditions, *Xcc* cells might produce enough *anteiso*-branch-chain 3-hydroxyacyl-ACP pools. Therefore, it might be the different substrate specificity of RpfF for various branched-chain 3-hydroxyacyl-ACP caused *Xcc* to produce different amounts of various DSF-family signals. But a more detail mechanism requires investigation.

## Author Contributions

K-HL cloned *ilvC* gene, constructed *ilvC* mutant strains, labeled *ilvC* mutant strain with 13C glucose and carried out experiments on the pathogenesis of *Xcc* strains. H-JD tested DSF signals produced by *Xcc* strains. W-BZ analyzed fatty acids composition of *Xcc* strains. Y-HY expressed and purified the *IlvC* protein. J-CM participated in the design of the study and helped to draft the manuscript. H-HW conceived of the study, and participated in its design and coordination and helped to draft the manuscript. All authors read and approve the final manuscript.

## Conflict of Interest Statement

The authors declare that the research was conducted in the absence of any commercial or financial relationships that could be construed as a potential conflict of interest.
