# Supplementary material for: Biological Functions of ilvC in Branched-Chain Fatty Acid Synthesis and Diffusible Signal Factor Family Production in Xanthomonas campestris
Source: Front Microbiol. 2017 Dec 12;8:2486. doi: 10.3389/fmicb.2017.02486 (PMC5733099; doi:10.3389/fmicb.2017.02486)
Supplement: Supplementary file 1 [file Data_Sheet_1.docx]

**Biological functions of *ilvC* in branched-chain fatty acid synthesis and diffusible signal factor family production in *Xanthomonas campestris***

Kai-Huai Li^1^, Yong-Hong Yu^2^, Hui-Juan Dong^1^, Wen-Bin Zhang^1^, Jin-Cheng Ma^1^, Hai-Hong Wang^1^*

Supplementary Tables

**Table S1. Bacterial strains, plasmids and primers used in this study**

| Strains | Relevant characteristics | References | |
| --- | --- | --- | --- |
| *E. coli* |  | |  |
| BL21(DE3) | F^-^ *dcm* *omp T* *hsdS*(r_B_^-^m_B_^-^) *gal* (λDE3) | | Lab collection |
| DH5α | F^-^ *deoR* *endA1 gyrA96 hsdR17*(r_K_^-^m_K_^+^) *recA1* *relA1* *supE*44 *thi-1* Δ(*lacZYA-argF*)*U*169(φ80*lacZ*ΔM15) | | Lab collection |
| S17-1 | Tp^r^ Sm^r^ *recA* *thi* *pro* *hsdR* (RP4-2 Tc::Mu Km::Tn*7*), λ*pir* | | Lab collection |
| *X. campestris pv. campestris* |  | |  |
| Xc1 | Rif^r^, Wild-type strain | |  |
| *Xcc ΔrpfBC* | Rif^r^, The *rpfB* and *rpfC* in-frame deletion double mutant of strain Xc1 | |  |
| *Xcc* KH1 | Rif^r^, The *ilvC* in-frame deletion mutant of strain Xc1 | | This study |
| *Xcc* KH2 | Rif^r^, The *ilvC* in-frame deletion mutant of strain *Xcc ΔrpfBC* | | This study |
| *Xcc* KH3 | Rif^r^, Gm^r^, *Xcc* KH1 strain carrying a single copy of *Xcc ilvC* encoded gene at *glmS* site constructed by Mini-Tn7 delivery plasmid pKH3. | | This study |
| *Xcc* KH4 | Rif^r^, Gm^r^, *Xcc* KH2 strain carrying a single copy of *Xcc ilvC* encoded gene at *glmS* site constructed by Mini-Tn7 delivery plasmid pKH3. | | This study |
| Plasmids |  | |  |
| pET28b(+) | Km^r^, T7 promoter-based expression vector | | Lab collection |
| pK18mobsacB | Km^r^, *sacB*-based gene replacement vector | | ([Schafer](file:///C:\\Users\\Administrator\\Desktop\\新建文件夹\\Table%20S1.docx#_ENREF_7) *[et al.](file:///C:\\Users\\Administrator\\Desktop\\新建文件夹\\Table%20S1.docx#_ENREF_7)*[, 1994](file:///C:\\Users\\Administrator\\Desktop\\新建文件夹\\Table%20S1.docx#_ENREF_7)) |
| pUC18-mini-Tn7T | Gm^r^, Mini-Tn7 delivery vector | | ([Choi](file:///C:\\Users\\Administrator\\Desktop\\新建文件夹\\Table%20S1.docx#_ENREF_2) *[et al.](file:///C:\\Users\\Administrator\\Desktop\\新建文件夹\\Table%20S1.docx#_ENREF_2)*[, 2005](file:///C:\\Users\\Administrator\\Desktop\\新建文件夹\\Table%20S1.docx#_ENREF_2)) |
| pTNS2 | Amp^r^, helper plasmid | | ([Choi et al., 2005](file:///C:\\Users\\Administrator\\Desktop\\新建文件夹\\Table%20S1.docx#_ENREF_2)) |
| pKH2 | Km^r^, *Xcc ilvC* in pET-28b | | This study |
| pKH3 | Gm^r^, *Xcc ilvC* in pUC18-mini-Tn7T | | This study |
| pKH5 | Km^r^, *Xcc ilvC* in-fame deletion fragment inserted to pK18mobscaB vector between EcoRI/HindIII sites | | This study |

**Table S2. Sequences of the PCR primers used in this work**

| Primer name | Primer sequence (5′ to 3′) | Digestion sites ^a^ |
| --- | --- | --- |
| *Xcc* ilvC NdeI | aattatgCATATGagcaacgacacccaacc | NdeI |
| *Xcc* ilvC BamH l | aattGGATCCagcacatcaatgatgagttg | BamHI |
| *Xcc* ilvC HindIII | aattAAGCTTcggacctttcggttaaacgg | HindIII |
| *Xcc* ilvC EcoRI | aattGAATTCcgtacgatgaccgaagtttcag | EcoRI |
| *Xcc* ilvC up1 | gtcagcacatctttcatgagcttcttctgcaccatgtc |  |
| *Xcc* ilvC down1 | catggtgcagaagaagctcatgaaagatgtgctgaccgac |  |
| *Xcc* ilvC HindIII | tataAAGCTTaccagaaagctgtgcttgacg | HindIII |

*^a^* Underlined, restriction endonuclease sites.

**Table S3. Media used in this study**

| Media | Compositions | References |
| --- | --- | --- |
| LB | 10 g/L Trypton, 5 g/L Yeast Extract, 10 g/L NaCl, pH 7.0 | ([Bertani, 1951](file:///C:\\Users\\Administrator\\Desktop\\新建文件夹\\Table%20S3.docx#_ENREF_1)) |
| NYG | 5 g/L Trypton, 3 g/L Yeast Extract, 20 g/L glycerol, pH 7.0 | ([Turner](file:///C:\\Users\\Administrator\\Desktop\\新建文件夹\\Table%20S3.docx#_ENREF_8) *[et al.](file:///C:\\Users\\Administrator\\Desktop\\新建文件夹\\Table%20S3.docx#_ENREF_8)*[, 1984](file:///C:\\Users\\Administrator\\Desktop\\新建文件夹\\Table%20S3.docx#_ENREF_8)) |
| NA | 5 g/L Trypton, 1 g/L Yeast Extract, 3 g/L beef extract, 10 g/L sucrose, pH 7.0 | ([Guo](file:///C:\\Users\\Administrator\\Desktop\\新建文件夹\\Table%20S3.docx#_ENREF_4) *[et al.](file:///C:\\Users\\Administrator\\Desktop\\新建文件夹\\Table%20S3.docx#_ENREF_4)*[, 2012](file:///C:\\Users\\Administrator\\Desktop\\新建文件夹\\Table%20S3.docx#_ENREF_4)) |
| XOLN | 0.7 g/L K_2_HPO_4_, 0.2 g/L KH_2_PO_4_, 1 g/L (NH_4_)_2_SO_4_, 0.1 g/L MgCl_2_, 0.01 g/L FeSO_4_, 0.001 g/L MnCl_2_, 2 g/L sucrose, 0.625g/L Trypton, 0.625 g/L Yeast Extract, pH 7.0 | ([Fu & Tseng, 1990](file:///C:\\Users\\Administrator\\Desktop\\新建文件夹\\Table%20S3.docx#_ENREF_3)) |
| XO | 0.7 g/L K_2_HPO_4_, 0.2 g/L KH_2_PO_4_, 1 g/L (NH_4_)_2_SO_4_, 0.1 g/L MgCl_2_, 0.01 g/L FeSO_4_, 0.001 g/L MnCl_2_, pH 7.0 | This study |
| XOS | 0.7 g/L K_2_HPO_4_, 0.2 g/L KH_2_PO_4_, 1 g/L (NH_4_)_2_SO_4_, 0.1 g/L MgCl_2_, 0.01 g/L FeSO_4_, 0.001 g/L MnCl_2_, 1 g/L sucrose, pH 7.0 | This study |
| XOG | 0.7 g/L K_2_HPO_4_, 0.2 g/L KH_2_PO_4_, 1 g/L (NH_4_)_2_SO_4_, 0.1 g/L MgCl_2_, 0.01 g/L FeSO_4_, 0.001 g/L MnCl_2_, 1 g/L glucose, pH 7.0 | This study |
| PBS | 0.7 g/L K_2_HPO_4_, 0.2 g/L KH_2_PO_4_, 0.1 g/L MgCl_2_, 0.01 g/L FeSO_4_, 0.001 g/L MnCl_2_, pH 7.0 | This study |
| PBSS | 0.7 g/L K_2_HPO_4_, 0.2 g/L KH_2_PO_4_, 0.1 g/L MgCl_2_, 0.01 g/L FeSO_4_, 0.001 g/L MnCl_2_, 2g/L sucrose，pH 7.0 | This study |
| Cabbage extracts | To obtain cabbage extracts, Chinese cabbage was minced using an electric juicer and cell debris was removed by centrifugation at 4000×g for 20 min at 4°C, and the supernatants were subjected to a 0.22 μm Mini-star filter unit and filtrates were saved as cabbage extracts. | This study |

**Table S4. Fatty acid composition of total lipid extracts from *Xcc* Xc1 grown in XOS medium supplemented with valine and isoleucine at 15°C** *^a^*

| Fatty acids (%) | XC1 | V+I (100μmol/L) | V+I (300μmol/L) |
| --- | --- | --- | --- |
| n-C_14:0_ 3-OH*^b^* | 3.01 ± 2.81 | 5.39 ± 1.18 | 6.43 ± 2.03 |
| *iso*-C_15:0_ | 5.64 ± 0.49 | 2.50 ± 0.13 | 1.97 ± 0.17 |
| *anteiso-*C_15:0_ | 7.50 ± 0.22 | 20.37 ± 1.52 | 18.85 ±1.39 |
| n-C_15:0_ | 0.18 ± 0.31 | 0.32 ± 0.28 | 0.30 ± 0.26 |
| *iso*-C_16:0_ | 3.74 ± 0.13 | 3.78 ± 0.21 | 3.08 ± 0.12 |
| n-C_16:1_ *cis*-9 | 33.68 ± 3.47 | 24.84 ± 0.21 | 23.12 ± 1.44 |
| n-C_16:0_ | 19.91 ± 2.56 | 12.19 ± 0.28 | 13.05 ± 0.98 |
| *iso*-C_17:1_ *cis*-9 | 8.01 ± 5.63 | 8.12 ± 0.84 | 9.43 ± 1.11 |
| *iso*-C_17:0_ | 3.90 ± 0.13 | 3.42 ± 0.26 | 2.78 ± 0.19 |
| *anteiso*-C_17:0_ | 0.35 ± 0.61 | 5.00 ± 0.28 | 4.28 ± 0.40 |
| n-C_17:1_ *cis*-10 | 0.41 ± 0.72 | 1.81 ± 0.08 | 1.63 ± 0.25 |
| n-C_18:1_ *cis*-11 | 10.99 ± 1.36 | 9.63 ± 1.84 | 11.68 ± 3.05 |
| n-C_18:0_ | 2.69 ± 0.41 | 2.63 ± 0.14 | 3.41 ± 0.65 |
|  |  |  |  |
| Total UFAs | 53.09 ± 2.25 | 44.40 ± 2.22 | 45.85 ± 2.15 |
| Total BCFAs | 21.13 ± 0.80 | 35.08 ± 1.64 | 30.96 ± 2.20 |
| iso-BCFAs | 13.28 ± 0.24 | 9.71 ±0.48 | 7.84 ± 0.43 |
| anteiso-BCFAs | 7.85 ± 0.66 | 25.37 ± 1.38 | 23.12 ± 1.78 |
| anteiso-/iso- | 0.59 ± 0.05 | 2.62 ± 0.15 | 2.95 ± 0.09 |

*^a^* Cells were grown in XOS medium supplemented with valine and isoleucine for 36 h at 15°C. Total lipids were extracted and transesterified to fatty acid methyl esters, and products identified by GC-MS. Values are percentages of total fatty acids and are means ± standard deviations of three independent experiments.

***^b^*** n-C_14:0_ 3-OH, 3-hydroxyltetradecanoic; *iso*-C_15:0_, 13-methyl-tetradecanoic acid; *anteiso*-C_15:0_, 12-methyl-tetradecanoic acid; n-C_15:0_, pentadecanoic acid; *iso*-C_16:0_, 14-methyl-pentadecanoic acid; n-C_16:1_, *cis*-9-hexadecenoic acid; n-C_16:0_, hexadecanoic acid; *iso*-C_17:1_ *cis*-9, *cis*-9-15-methyl-hexadecenoic acid; *iso*-C_17:0_, 15-methyl-hexadecanoic acid; *anteiso*-C_17:0_, 14-methyl-hexadecanoic acid; n-C_17:1_ *cis*-10, *cis*-10-hexadecenoic acid; n-C_18:1_ *cis*-11, *cis*-11-octadecenoic acid; n-C_18:0_, octadecanoic acid. UFA, unsaturated fatty acid; BCFA, branched-chain fatty acid.

**Supplementary Figures**

**Figure S1**

**Figure S1.** Alignment of *Xcc* and *P. aeruginosa* IlvC. Pa, *P. aeruginosa*; Xc, *Xcc*. Gray boxes I, II, III, IV and V, conserved regions; red stars, positions of active site residues. Alignment was with Clustal W based on identical residues.

**Figure S2**

**Figure S2.** A. Strategy for isolation of *Xcc* KH1 mutant strain. B. Genetic organization of the *ilvC* region in *Xcc* Xc1 (a) or *Xcc* KH1 (b). C. PCR analysis of genomic DNA from strains in (B). Abbreviations: CH, chromosome; Up, upstream fragment of *Xcc ilvC*; Dn, downstream fragment of *Xcc ilvC*.

**Figure S3**

**Figure S3.** A. Mutant strain *Xcc* KH1 in XOS liquid medium supplemented with KIV and KMV mixtures at 30°C. B. Mutant strain *Xcc* KH1 in XOS liquid medium supplemented with KIV and KMV mixtures at 15°C. Filled circles, no supplement of KIV and KMV; filled squares, 100 μmol/L KIV and KMV mixture; filled triangles, 300 μmol/L KIV and KMV mixture; empty circles, 500 μmol/L KIV and KMV mixture. After growth, OD_600_ was monitored using the Bioscreen-C Automated Growth Curves Analysis System (OY Growth Curves FP-1100-C, Helsinki, Finland).

**Figure S4**

**Figure. S4. Mass spectra of ^13^C-labeled FAMES.** A. Mass spectra of ^13^C-labeled methyl *iso*-pentadecanoate from wild type *Xcc* Xc1. B. Mass spectra of ^13^C-labeled methyl *iso*-pentadecanoate from mutant KH1. Parent molecular ions ([M^+^]) and select fragment ions are indicated, including for the McLafferty ion.

**Figure S5**

**Figure S5.** **DSF-family signals from *ilvC* deletion mutant**. A. DSF signals from *Xcc ilvC* deletion mutant KH2 in XOLN medium (Table S3). Supernatants of 50 mL KH2 grown in XOLN medium for 36 h were collected and DSF signals detected. B and C. DSF signals produced by resting cells of KH2 in PBSS medium supplemented with branched-chain 2-keto acids. DSF signals were extracted and detected. Error bars, mean ± standard deviation (n = 3). * P < 0.05, ** P < 0.01, *** P < 0.001, assessed with one-way ANOVA. All experiments were repeated three times with similar results.
